# Combined Toxicity of Insecticides and Fungicides Applied to California Almond Orchards to Honey Bee Larvae and Adults

**DOI:** 10.3390/insects10010020

**Published:** 2019-01-08

**Authors:** Andrea Wade, Chia-Hua Lin, Colin Kurkul, Erzsébet Ravasz Regan, Reed M. Johnson

**Affiliations:** 1 Department of Biochemistry and Molecular Biology, College of Wooster, Wooster, OH 44691, USA; awade17@wooster.edu (A.W.); eregan@wooster.edu (E.R.R.); 2 Department of Entomology, The Ohio State University, Columbus, OH 43210, USA; lin.724@osu.edu; 3 Department of Microbiology, The Ohio State University, Columbus, OH 43210, USA; kurkul.4@osu.edu; 4 Department of Entomology, The Ohio State University, Wooster, OH 44691, USA

**Keywords:** synergism, *Apis mellifera*, Altacor, Intrepid, Dimilin, Pristine, Tilt, Rovral, pollination

## Abstract

Beekeepers providing pollination services for California almond orchards have reported observing dead or malformed brood during and immediately after almond bloom—effects that they attribute to pesticide exposure. The objective of this study was to test commonly used insecticides and fungicides during almond bloom on honey bee larval development in a laboratory bioassay. In vitro rearing of worker honey bee larvae was performed to test the effect of three insecticides (chlorantraniliprole, diflubenzuron, and methoxyfenozide) and three fungicides (propiconazole, iprodione, and a mixture of boscalid-pyraclostrobin), applied alone or in insecticide-fungicide combinations, on larval development. Young worker larvae were fed diets contaminated with active ingredients at concentration ratios simulating a tank-mix at the maximum label rate. Overall, larvae receiving insecticide and insecticide-fungicide combinations were less likely to survive to adulthood when compared to the control or fungicide-only treatments. The insecticide chlorantraniliprole increased larval mortality when combined with the fungicides propiconazole or iprodione, but not alone; the chlorantraniliprole-propiconazole combination was also found to be highly toxic to adult workers treated topically. Diflubenzuron generally increased larval mortality, but no synergistic effect was observed when combined with fungicides. Neither methoxyfenozide nor any methoxyfenozide-fungicide combination increased mortality. Exposure to insecticides applied during almond bloom has the potential to harm honey bees and this effect may, in certain instances, be more damaging when insecticides are applied in combination with fungicides.

## 1. Introduction

The honey bee (*Apis mellifera*) is heavily relied upon by modern agriculture for pollination services. Almonds are highly dependent on insect pollination [1] and as a result, growers of California almonds rented 1.48 million colonies of honey bees in 2017 at a cost of over 253 million dollars [2]. A number of honey bee colonies rented for almond pollination in California in 2014 were impacted by honey bee die-offs. Reportedly, 40% of colonies in almond orchards experienced adult honey bee deaths or dead and deformed brood, and 20% of colonies were completely dead [3].

**Figure 1:**
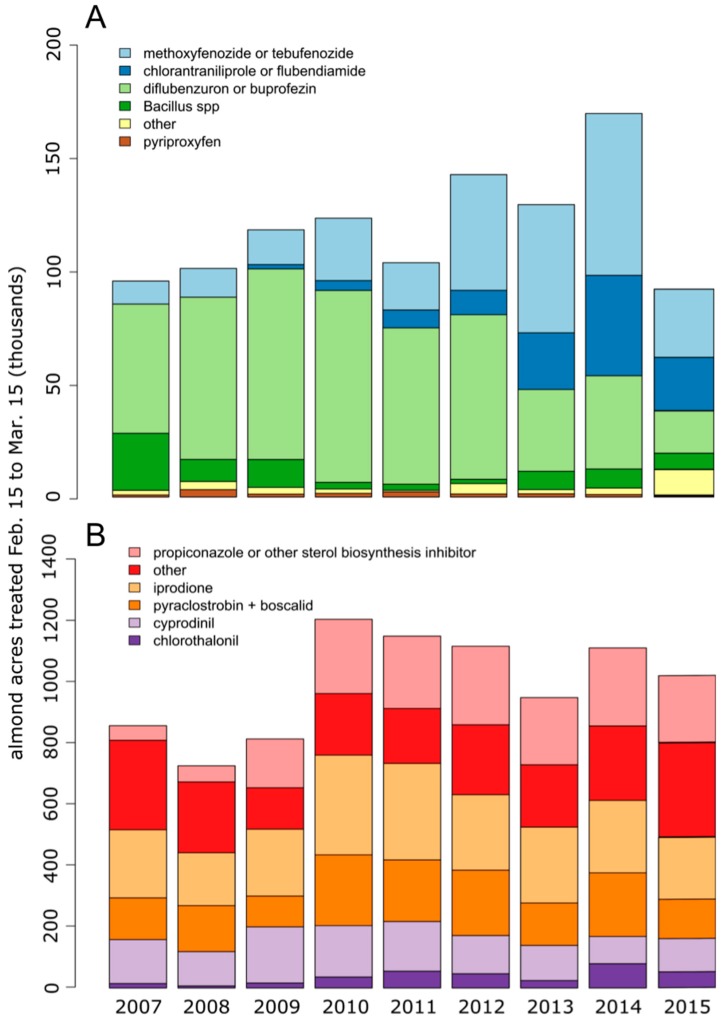
Insecticide (**A**) and fungicide (**B**) use on California almonds for the years 2007–2015 during the blooming period (15 February–15 March). Data downloaded from the California Pesticide Information Portal [4].

Pesticide use data reported by the California Pesticide Information Portal (CALPIP) [4] indicates that a range of insecticides and fungicides are applied to almond orchards during the blooming period (Figure 1). Insecticides that do not carry label language prohibiting their application when bees are present, such as chlorantraniliprole, methoxyfenozide, and diflubenzuron, have been applied during bloom to control the peach twig borer (*Anarsia lineatella*) [5]. These insecticides work through varying modes of action targeting developmental processes and muscle function.

Chlorantraniliprole is a diamide insecticide that acts as a ryanodine receptor modulator [6] and is classified by the Insecticide Resistance Action Committee (IRAC) as a Group 28 mode of action [7]. It is marketed under the trade name Altacor, which is labeled for application to almonds during bloom [8]. Previous work has found that chlorantraniliprole demonstrates low intrinsic toxicity to honey bees [9]. A second diamide, flubendiamide, has also been applied to almonds during bloom, but its use was discontinued in 2016 due to possible adverse effects on aquatic invertebrates [10].

Methoxyfenozide is a diacylhydrazine-based ecdysone receptor agonist insecticide (IRAC Group 18) marketed under the trade name Intrepid 2F. It binds to ecdysone receptors of insects, causing premature molting of larvae and death [11]. Methoxyfenozide is selective for lepidopteran pests and is registered as a “Reduced Risk Pesticide” with the United States Environmental Protection Agency (US EPA) because of its low impact on bees and other beneficial insects [12,13]. Therefore, it may be applied to almonds during bloom [14]. However, field-relevant spray applications have been found to reduce adult worker survival over 10 days [15].

Diflubenzuron, marketed under the trade name Dimilin 2L, is an insect growth regulator (IRAC Group 15) that disrupts the normal molting of insect larvae through the inhibition of chitin synthesis [16]. Diflubenzuron is used to control a number of lepidopteran pests on almonds and has been recommended for application during bloom [17]. Previous studies have demonstrated the low acute toxicity of diflubenzuron to adult honey bees [18,19,20,21], though physiological effects and impaired learning have been reported [22,23,24]. Diflubenzuron has also been shown to reduce brood production in whole colonies [18,20,25,26,27] and negatively affect the survival of immature queens [28].

Fungicides generally display low acute toxicity to honey bees, but may enhance the toxicity of insecticides when applied together in a tank-mix [29]. Pesticide applications reported in the CALPIP database indicate that more than 95% of insecticide applications made on almonds trees during bloom are applied at the same time as fungicides. Fungicides commonly applied during the blooming period include iprodione (Rovral 4 and Iprodione 2SE), a formulated mix of boscalid and pyraclostrobin (Pristine), and sterol biosynthesis-inhibiting (SBI) fungicides, including propiconazole (Tilt). 

Iprodione has been shown to affect larval survival and cause malformations during development [30], but demonstrated no effect on adults [31]. The formulated fungicide Pristine has been shown to reduce mitochondrial function [32] in adult workers, but had no effect on development of immature queens [28]. A tank-mix combination of Pristine and iprodione may, however, cause elevated adult worker mortality [33]. The sterol biosynthesis-inhibiting (SBI) class of fungicides, in particular, has been demonstrated to increase the toxicity to honey bees of certain pyrethroid, neonicotinoid, and organophosphate insecticides [34,35,36,37,38,39], as well as the toxicity of the natural toxin quercetin found in pollen [40]. 

The unexpectedly high number of honey bee deaths observed when certain insecticides and SBI fungicides are co-applied can be more specifically characterized as synergistic toxicity, which is the toxicity of a chemical combination that is greater than that predicted from studies of isolated chemical constituents [41]. The synergistic toxicity between previously studied insecticides and SBI fungicides in honey bees is likely due to honey bee cytochrome P450 monooxygenase (P450) enzyme inhibition. SBI fungicides target CYP51 in fungi, a family of P450 enzymes involved in ergosterol biosynthesis [42]. Although these fungicides are intended to inhibit the fungal CYP51 family P450s, research on the selectivity of five different SBI fungicides revealed that each demonstrated non-selective inhibition of P450s [43], which likely results in drug–drug interactions in vivo.

P450s are monooxygenase enzymes that have evolved to serve many roles and can be found in all organisms [44]. While P450s are involved in biosynthetic pathways for hormones, they are also involved in detoxification pathways in many organisms, including honey bees. P450s often perform the first oxidative step in the detoxification of foreign compounds [45]. Certain families of insect P450s are known for pesticide metabolism and are responsible for insecticide resistance in crop pests. Honey bee P450s in the CYP9Q subfamily have been identified as enzymes involved in the metabolism of a pyrethroid and an organophosphate acaricide used in hives [46]. Iwasa et al. [37] demonstrated that honey bees fed SBI fungicides were incapable of metabolizing some neonicotinoids. These findings are evidence of the central role played by P450s in honey bee detoxification of insecticides. In comparing the genome of the honey bee with those of other insects, the honey bee genome encodes for fewer *P450* genes, with 46 honey bee *P450* genes in total [47]. It has been suggested that this deficit could make them vulnerable to synergistic interactions between pesticides [45]. For this reason, the testing of pesticide combinations on honey bees is particularly important. 

This study investigates the effects of the SBI fungicide propiconazole, as well as other fungicides commonly applied to almonds during bloom, including iprodione (Rovral) and the combination of pyraclostrobin and boscalid (Pristine), on the toxicity of the insecticides chlorantraniliprole, methoxyfenozide, and diflubenzuron to larval honey bees reared using an in vitro method [48] with adult emergence as the measurement endpoint. Topical application of chlorantraniliprole and propiconazole to adult bees was also performed to generate dose response curves to determine adult LD_50_ values [49] for risk interpretation using the BeeRex model produced by the US EPA [50].

## 2. Materials and Methods

### 2.1. Chemicals

Technical grade pesticides were used for all bioassays (>95% purity, Sigma Aldrich, St. Louis, MI, USA). Serial dilutions in acetone were used to create test solutions for adult or larval treatment. Relative concentrations of the insecticides and fungicides used were determined based on the ratio of the maximum label rate of active ingredient for application to almonds.

### 2.2. Honey Bees

Honey bee colonies headed by young, commercially produced, non-sister Italian or New World Carniolan queens were maintained at The Ohio State University apiaries in Wooster and Columbus and managed according to standard beekeeping practice. Colonies were treated for *Varroa destructor* mites using formic acid or oxalic acid according to label instructions as needed. No synthetic miticides that may accumulate in beeswax [51] had ever been used in hive equipment in prior years. A total of 15 colonies of these were used in adult and larval studies carried out from late April through early September in 2016 and 2017.

### 2.3. In vitro Larval Tests

**Figure 2:**
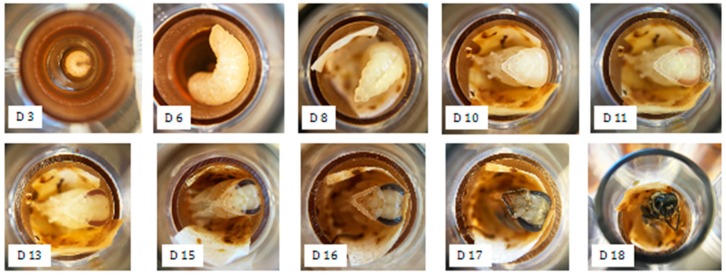
Photos of honey bee larval development reared in vitro. “D” corresponds to days after larval transfer.

In vitro larval rearing bioassays (Figure 2) were performed using the Aupinel method [52] as modified by Schmehl et al. [48]. Briefly, young larvae, less than 24 h old, were grafted into 48-welled polystyrene microplates (Corning Life Sciences, Oneonta, NY, USA) lined with Brown Cell Cups (Mann Lake Ltd., Hackensack, MN, USA), where they were kept in an incubated desiccator at 35 °C and 95% humidity. The larvae were fed a larval diet until pupation, when they were transferred to fresh microplates and moved to a second desiccator maintained at 75% humidity. The larval diet consisted of distilled water, royal jelly (Stakich, Troy, MI, USA), glucose, fructose, and yeast extract (Fisher Scientific, Columbus, OH, USA). Larvae were fed a diet containing a test treatment or solvent control on day 4 of the larval feeding schedule. Pesticides dissolved in acetone were incorporated into the larval diet at 2% total volume. Insecticide treatments consisted of 1 μg chlorantraniliprole, 2.25 μg methoxyfenozide, and 2.28 μg diflubenzuron per larva. Fungicide treatments consisted of 2.25 μg propiconazole, 5.05 μg iprodione, and the combination of 4.68 μg boscalid and 2.37 μg pyraclostrobin per larva. Insecticide-fungicide combination treatments, a negative acetone-only control, and a positive control containing 5 μg dimethoate per larva were included throughout the study in accordance with the Aupinel method [52]. At least three replicates were performed for each treatment. Each treatment replicate consisted of between 10 and 16 larvae grafted from two to three different colonies. In total, 1417 larvae were taken from 12 colonies for in vitro testing. 

### 2.4. Adult tests

Topical dose response bioassays were performed on 3-day-old adult worker bees. Frames of late stage capped brood were taken from four colonies and kept in a dark humidified (60–80% RH) incubator at 34 °C. Bees were taken from four different colonies. Newly emerged bees were brushed from frames and placed in groups of approximately 20 in paper cups (177 cm^3^; Uniq 6 oz. Ice Cream Cup, FrozenDessertSupplies.com, Gilbert, AZ, USA). Cups were covered with cotton cheese cloth (Grade 40; Raglady.com, Stevensville, MD) and secured with rubber bands. Each cup of bees was returned to the incubator and provisioned with water in a punctured microcentrifuge tube (1.5 mL; Fisher Scientific) and sugar candy made from finely ground granulated sugar and heavy sugar syrup (2:1 sucrose in water *w*/*w*).

After three days of feeding, bees were topically dosed with 5 μl acetone solutions containing insecticide, fungicide, a combination, or acetone containing no test chemical. A 50-μl glass micropipette (Hamilton 705SNR, Reno, NV, USA) fitted in a repeating dispenser (Hamilton PB-600) was used to apply doses to the dorsal surface of the bee thorax. Seven chlorantraniliprole doses ranging from 0.005 μg to 5 μg per bee were administered. A single dose at the solubility limit of methoxyfenozide in acetone, 25 μg per bee, was used. Seven combination treatments containing chlorantraniliprole (0.005 to 5.0 μg per bee) with propiconazole (0.011 to 11.260 μg per bee) were administered. Preliminary bioassays confirmed that propiconazole alone up to 11.3 μg per bee did not result in significant mortality. Each treatment replicate was accompanied by an acetone solvent control group. At least four dose response replicates consisting of eight cups were performed for chlorantraniliprole and the combination treatments, testing a total of 1347 adult bees. Three replicates of the methoxyfenozide treatment were performed. Mortality was recorded 48 h after topical treatment.

### 2.5. Statistical Analysis

Results from larval rearing bioassays were analyzed with a generalized linear mixed model in SAS (PROC GLIMMIX, version 9.4; SAS Institute Inc. Cary, NC, USA). A binomial distribution was used as the response variable was binary; live or dead. Treatment was used as the fixed effect and grafting day as random effect. Post hoc pairwise comparisons of survival between treatments were performed using the Least Squares Means difference.

Mortality data from adult bee dose response bioassays was analyzed using generalized linear models (GLMs) with a log-probit transformation to calculate LD_50_ values and 95% confidence intervals [38,53]. Pairwise tests were used to compare treatments and assess interactive effects between test chemical combinations. A test of parallelism was used to assess differences in the slope of the dose-response. A test of equality was used to assess differences between dose-response in one treatment relative to another. Statistical analysis of the results from adult assays was performed in R (version 3.4.2, R Foundation for Statistical Computing, Vienna, Austria).

### 2.6. BeeREX Model

BeeREX (version 1.0) is a model developed by the US EPA Office of Pesticide Program’s Environmental Fate and Effects Division [50] as a Tier 1 screening tool to assess risks specifically posed by pesticides to honey bees. BeeREX risk quotients for acute topical chlorantraniliprole as well as topical chlorantraniliprole combined with propiconazole were calculated using LD_50_ values generated in this study.

## 3. Results

### 3.1. In vitro Larval Tests

Honey bee larvae raised in the lab were fed diets containing an insecticide or fungicide alone, or a combination of an insecticide and a fungicide at ratios simulating a real-world tank-mix. The insecticides chlorantraniliprole, methoxyfenozide, and diflubenzuron were tested, as well as the fungicides propiconazole, iprodione, and the combination of boscalid and pyraclostrobin (Table S1). Larvae in the solvent control and positive dimethoate control groups survived to adult emergence 73.8% and 50.2% of the time, respectively. Larvae treated with the insecticides chlorantraniliprole, methoxyfenozide, and diflubenzuron demonstrated 62.8%, 61.4%, and 12.4% survival to adult emergence, respectively. Larvae treated with the fungicides propiconazole, iprodione, and boscalid and pyraclostrobin demonstrated 68.1% 63.3%, and 67.3% survival to adult emergence, respectively.

**Figure 3:**
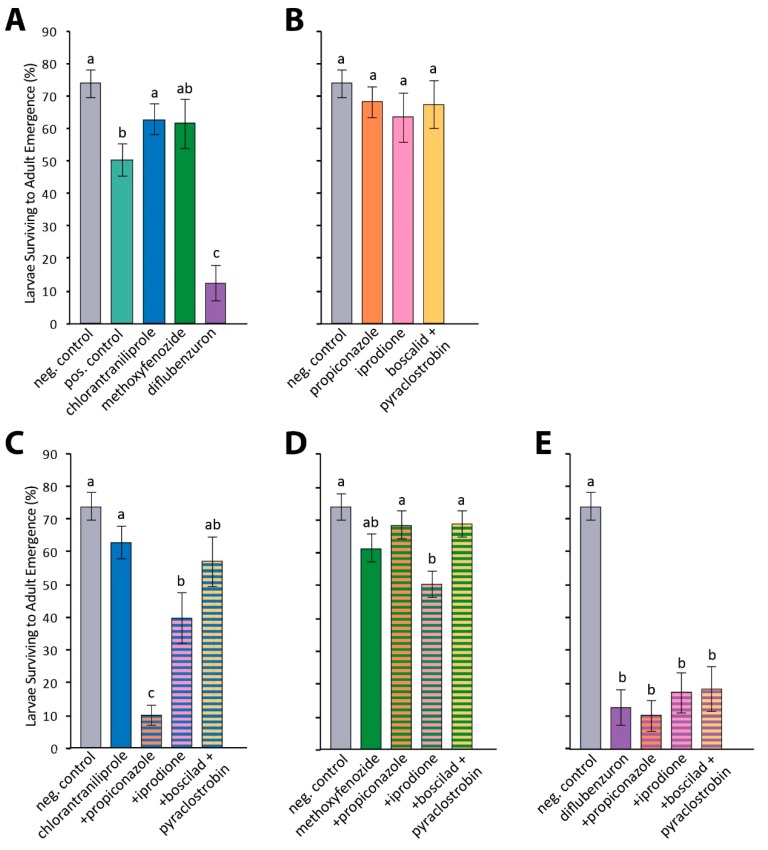
Effects of insecticides, fungicides, and combination treatments on larval survival. Average percent larvae surviving to adult emergence is shown, with error bars displaying standard error. Treatments with different letters indicate significantly different levels of adult emergence (*p* < 0.05). Negative solvent control (N = 130) and insecticide-only results are reproduced across subplots. (**A**) Insecticide treatments (dimethoate positive control N = 130; chlorantraniliprole N = 131; methoxyfenozide N = 114; diflubenzuron N = 63); (**B**) fungicide treatments (propiconazole N = 63; iprodione N = 47; boscalid and pyraclostrobin N = 47); (**C**) chlorantraniliprole combination treatments (+propiconazole N = 146; +iprodione N = 48; +boscalid and pyraclostrobin N = 63); (**D**) methoxyfenozide combination treatments (+propiconazole N = 79; +iprodione N = 63; +boscalid + pyraclostrobin N = 63); and (**E**) diflubenzuron combination treatments (+propiconazole N = 63; +iprodione N = 63; +boscalid and pyraclostrobin N = 47).

Treatment had a significant effect on larval survival (F_16,71_ = 11.58; *p* < 0.0001). The treatment of larvae with chlorantraniliprole or methoxyfenozide alone did not result in a significant decrease in adult emergence compared to the negative solvent control, while diflubenzuron did cause significantly reduced adult emergence (*p* < 0.001) (Figure 3A). Each fungicide tested alone did not result in a significant decrease in adult emergence compared to the solvent control (Figure 3B). Larvae treated with chlorantraniliprole in combination with propiconazole, iprodione, and boscalid plus pyraclostrobin had adult emergences of 10.1%, 39.7%, and 57.0%, respectively (Figure 3C). Combining propiconazole or iprodione with chlorantraniliprole significantly reduced adult emergence compared to larvae fed a diet containing chlorantraniliprole alone (*p* < 0.01 for both comparisons). Combination treatments of methoxyfenozide with fungicides did not result in a significant change in emergence compared to methoxyfenozide alone, though the combination of methoxyfenozide and iprodione resulted in reduced emergence relative to the control group (*p* < 0.05; Figure 3D). Combination treatments of diflubenzuron with any of the three fungicides did not significantly reduce adult emergence compared to diflubenzuron alone, but all larvae treated with diflubenzuron, alone or in combination, were less likely to emerge as adults than larvae treated with the solvent control (*p* < 0.001; Figure 3E). 

### 3.2. Adult Topical Tests

**Figure 4:**
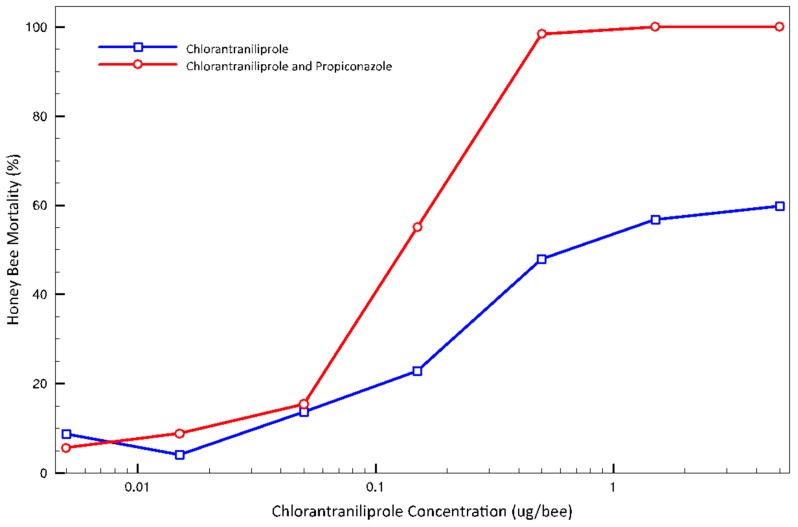
Dose-response curves for topical treatment of adult worker honey bees with chlorantraniliprole (N = 697) or chlorantraniliprole mixed with propiconazole (N = 476) at a 1:2.25 ratio.

The combination treatment of chlorantraniliprole and propiconazole resulted in higher bee mortality compared to the treatment of chlorantraniliprole alone (Figure 4), with the combination resulting in a 7.2-fold increase in toxicity (chlorantraniliprole LD_50_ = 0.706 (0.275–2.951 95% CI) μg/bee; chlorantraniliprole LD_50_ with propiconazole = 0.098 (0.046–0.206 95% CI) μg/bee). The equality deviance between treatments’ GLMs was 5.229 (*p* < 0.001).

## 4. Discussion

During almond pollination honey bees may be exposed to a variety of insecticides and fungicides, most of which have not been tested in combination with each other for synergistic effects. This study aimed to determine whether the most common insecticides and fungicides used on California almonds during bloom exhibit synergistic toxicity to honey bee larvae and adults when combined.

Diflubenzuron continues to be applied to California almonds during bloom, although its use has decreased in the past several years (Figure 1). Previous studies have demonstrated that diflubenzuron is of low toxicity to adult bees, yet it is capable of producing negative impacts on brood production [18,20,21,25,26,27]. The significant decrease in larval survival observed in the diflubenzuron-treated larvae in this study suggests that diflubenzuron, regardless of its tank-mix companions, does pose a risk to honey bee larvae.

The addition of the SBI fungicide propiconazole to chlorantraniliprole resulted in a substantial increase in toxicity to both adult and larval honey bees compared to chlorantraniliprole alone. The synergistic toxicity observed with chlorantraniliprole is likely caused by propiconazole inhibiting P450 enzymes, which serve to detoxify this insecticide in a similar way to how other SBI fungicides enhance the toxicity of other insecticide classes [38]. This suggests that honey bee P450s may play a critical role in chlorantraniliprole tolerance in honey bees. Elucidating the role of P450s in diamide detoxification in bees should be a priority for future research.

The BeeREX 1.0 model [50] for Tier 1 risk assessment allows further interpretation of the toxicity data generated. BeeREX allows for the calculation of Risk Quotients (RQ) by relating the LD_50_ values and the maximum field application rate. The Level of Concern (LOC) for acute honey bee exposure has been set at an RQ < 0.4, a level at which minimal harm to bees in field applications is expected [54]. Using BeeREX, we determined that chlorantraniliprole applied at the maximum label rate for almonds (0.099 lbs./acre) has an RQ of 0.37. A simulated tank-mix combination of chlorantraniliprole and propiconazole, each at their maximum labeled application rates, generates an RQ of 2.73, which exceeds the LOC and indicates that this insecticide-fungicide combination may pose a risk to bee health. 

The BeeREX model can also be used to estimate larval honey bee exposure to chlorantraniliprole to be 1.35 μg at the maximum label-recommended application rate. In larval rearing bioassays, 1 μg chlorantraniliprole with 2.25 μg propiconazole resulted in an average of 89.1% larval mortality. Pollen contaminated with chlorantraniliprole and propiconazole that is brought back to hives has the potential to kill immature bees. 

## 5. Conclusions

This study demonstrates the risks that pesticides used in almond orchards during bloom pose to honey bees. The larval toxicity of diflubenzuron as well as the adult and larval toxicity of chlorantraniliprole when combined with an SBI fungicide, propiconazole, could explain the sporadic losses observed by beekeepers in almond orchards. The findings presented here demonstrate how pesticides that initially appear to be safe for honey bees may demonstrate toxicity to worker adults and larvae in field-relevant tank-mix combinations. 

## Supplementary Materials

The following are available online at http://www.mdpi.com/2075-4450/10/1/20/s1, Table S1: Raw bioassay data.
